# Supplementary material for: [68Ga]Ga-DFO-c(RGDyK): Synthesis and Evaluation of Its Potential for Tumor Imaging in Mice
Source: Int J Mol Sci. 2021 Jul 9;22(14):7391. doi: 10.3390/ijms22147391 (PMC8306578; doi:10.3390/ijms22147391)

Supplementary Materials

[^{68}Ga]Ga-DFO-c(RGDyK): synthesis and evaluation of its potential for tumor imaging in mice

Sona Krajcovicova[1], Andrea Daniskova[2], Katerina Bendova[2], Zbynek Novy[2], Miroslav Soural[1,2*], Milos Petrik[2*]

[1] Department of Organic Chemistry, Faculty of Science, Palacky University, 77900 Olomouc, Czech Republic

[2] Institute of Molecular and Translational Medicine, Faculty of Medicine and Dentistry, Palacky University, 77900 Olomouc, Czech Republic

Analytical data

*Protected cyclized pentapeptide **8***

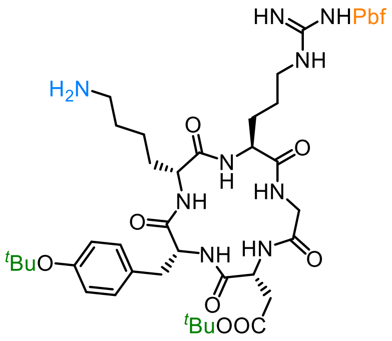

8

White solid (157 mg, 76% yield calculated from loading of **6** (0.7 mmol/g)).

^{1}H NMR (500 MHz, DMSO-d_6): δ 8.36 – 8.30 (m, 2H), 8.07 (t, J = 7.8 Hz, 1H), 7.98 (d, J = 8.1 Hz, 1H), 7.05 – 7.01 (m, 2H), 6.83 – 6.78 (m, 2H), 6.42 (br s, 1H), 4.61 – 4.56 (m, 1H), 4.41 – 4.37 (m, 1H), 4.12 – 4.09 (m, 1H), 4.06 – 3.98 (m, 2H), 3.27 – 3.24 (m, 3H), 3.22 – 3.19 (dd, J = 15.2, 3.9 Hz, 2H), 3.00 – 2.95 (m, 3H), 2.93 (s, 2H), 2.91 – 2.85 (m, 1H), 2.72 – 2.68 (m, 1H), 2.64 – 2.62 (m, 2H), 2.58 – 2.52 (m, 1H), 2.44 (s, 3H),

2.38 (s, 3H), 2.28 (dd, J = 15.5, 6.3 Hz, 1H), 1.97 (s, 3H), 1.90 – 1.87 (m, 2H), 1.77 (s, 2H), 1.71 – 1.68 (m, 2H), 1.37 (s, 3H), 1.32 (s, 9H), 1.23 (s, 9H) ppm.

^{13}C NMR (126 MHz, DMSO-d_6): δ 173.2, 171.8, 171.0, 170.4, 169.8, 169.3, 169.1, 157.4, 156.0, 153.3, 137.2, 134.1, 132.1, 131.3, 129.5, 124.2, 123.3, 116.2, 86.2, 79.8, 77.5, 54.3, 54.1, 51.9, 48.8, 47.3, 47.3, 45.8, 45.8, 43.2, 42.4, 36.7, 36.4, 30.9, 28.5, 28.2, 27.6, 27.7, 25.9, 25.8, 25.7, 25.6, 25.6, 22.2, 18.9, 17.5, 12.2 ppm.

HRMS (ESI): m/z calcd for $C_{48}H_{74}N_{11}O_9S_1$ $[M+H]^+$ = 984.5220, found $[M+H]^+$ = 984.5223.

*Final cRGD-deferoxamine conjugate **9***

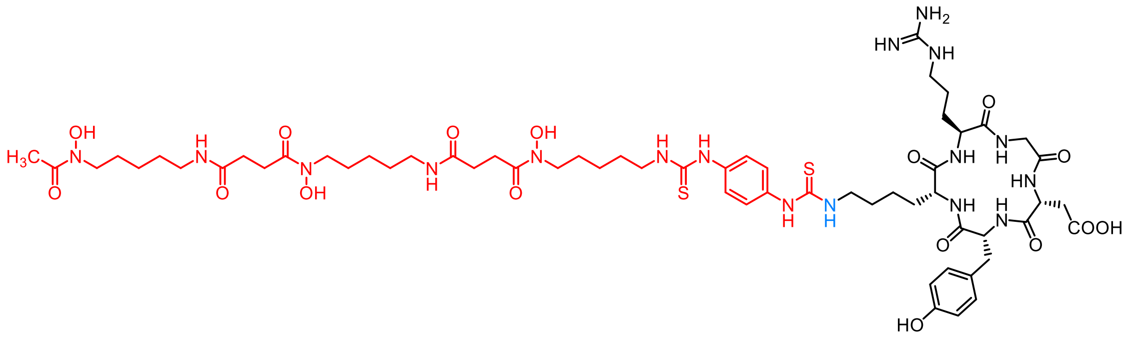

9

Pale brown solid (6 mg, 30% yield).

^{1}H NMR (500 MHz, DMSO-d_6): δ 9.64 – 9.62 (m, 3H), 9.38 (br s, 1H), 9.10 (br s, 1H), 8.30 – 8.28 (m, 1H), 8.22 – 8.07 (m, 3H), 7.94 – 7.91 (m, 2H), 7.79 – 7.73 (m, 2H), 7.67 – 7.62 (m, 2H), 7.41 – 7.36 (m, 2H), 7.34 – 7.28 (m, 2H), 7.07 – 7.04 (m, 1H), 6.96 – 6.92 (m, 2H), 6.63 – 6.57 (m, 2H), 4.56 – 4.51 (m, 1H), 4.48 – 4.46 (m, 1H), 4.35 – 4.28 (m, 2H), 4.16 (dd, J = 16.3, 8.9 Hz, 1H), 3.52 – 3.40 (m, 11H), 3.11 – 3.07 (m, 2H), 3.11 – 2.98 (m, 4H), 2.65 – 2.54 (m, 8H), 2.29 – 2.25 (m, 4H), 1.96 (s, 3H), 1.73 – 1.68 (m, 1H), 1.66 – 1.57 (m, 4H), 1.56 – 1.42 (m, 13H), 1.40 – 1.35 (m, 4H), 1.29 – 1.17 (m, 9H) ppm.

^{13}C NMR (126 MHz, DMSO-d_6): δ 172.0, 171.2, 170.6, 169.8, 156.6, 155.5, 154.7, 154.4, 129.8, 128.8, 127.6, 123.0, 114.8, 54.9, 53.3, 51.1, 48.7, 47.0, 44.7, 44.3, 43.8, 43.3, 42.6, 38.3, 31.5, 29.8, 28.7, 28.2, 27.5, 27.1, 26.5, 26.0, 23.4, 23.0, 21.0, 20.3 ppm.

Due to low signal-to-noise ratio not all carbon signals were detected.

HRMS (ESI): m/z calcd for $C_{60}H_{94}N_{16}O_{17}S_2$ $[M+H]^+$ = 1372.6500, found $[M+H]^+$ = 1372.6500.

Spectral data

*UHPLC/UV traces of compounds **2–6** after cleavage from the resin*

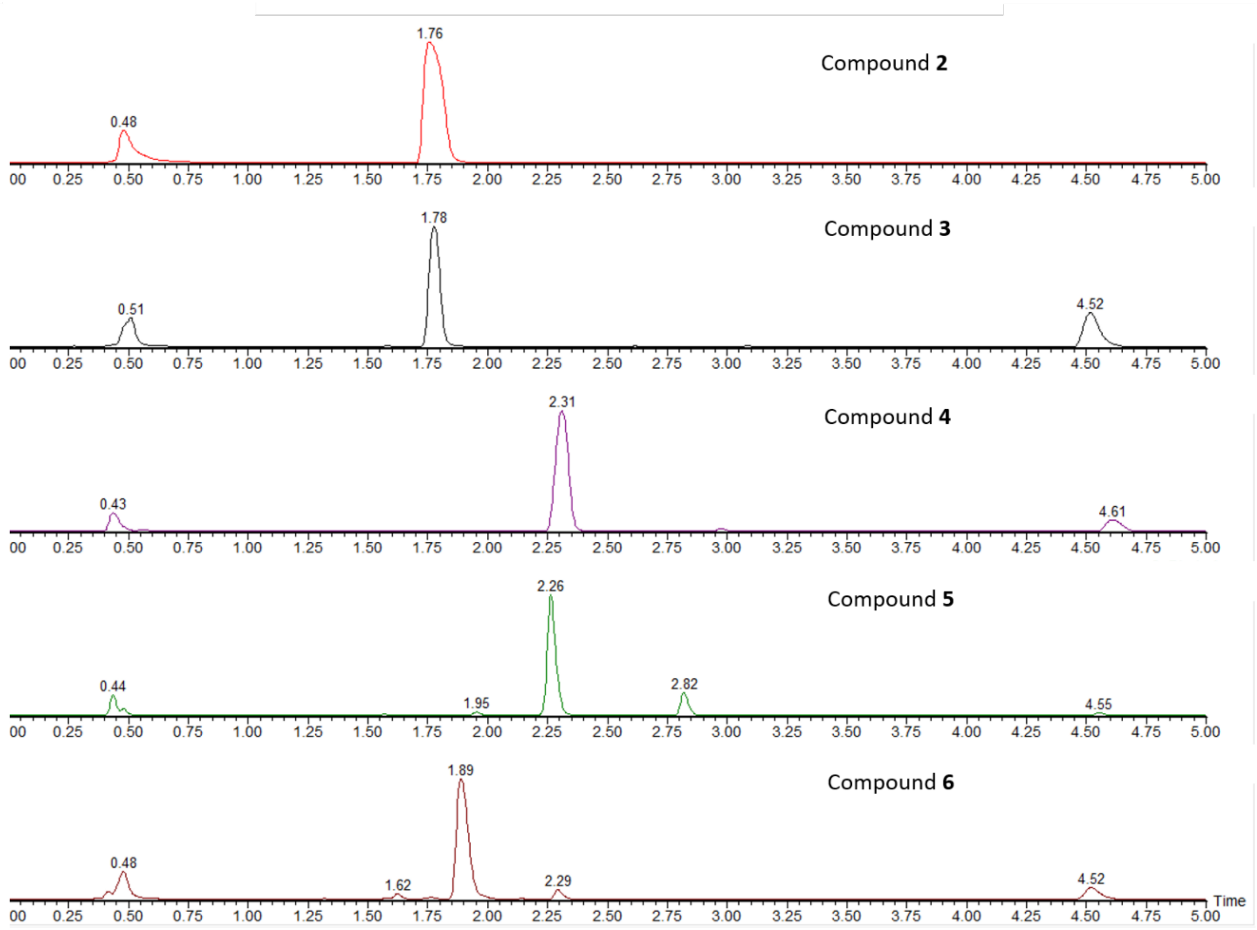

Protected cyclized pentapeptide ***8***

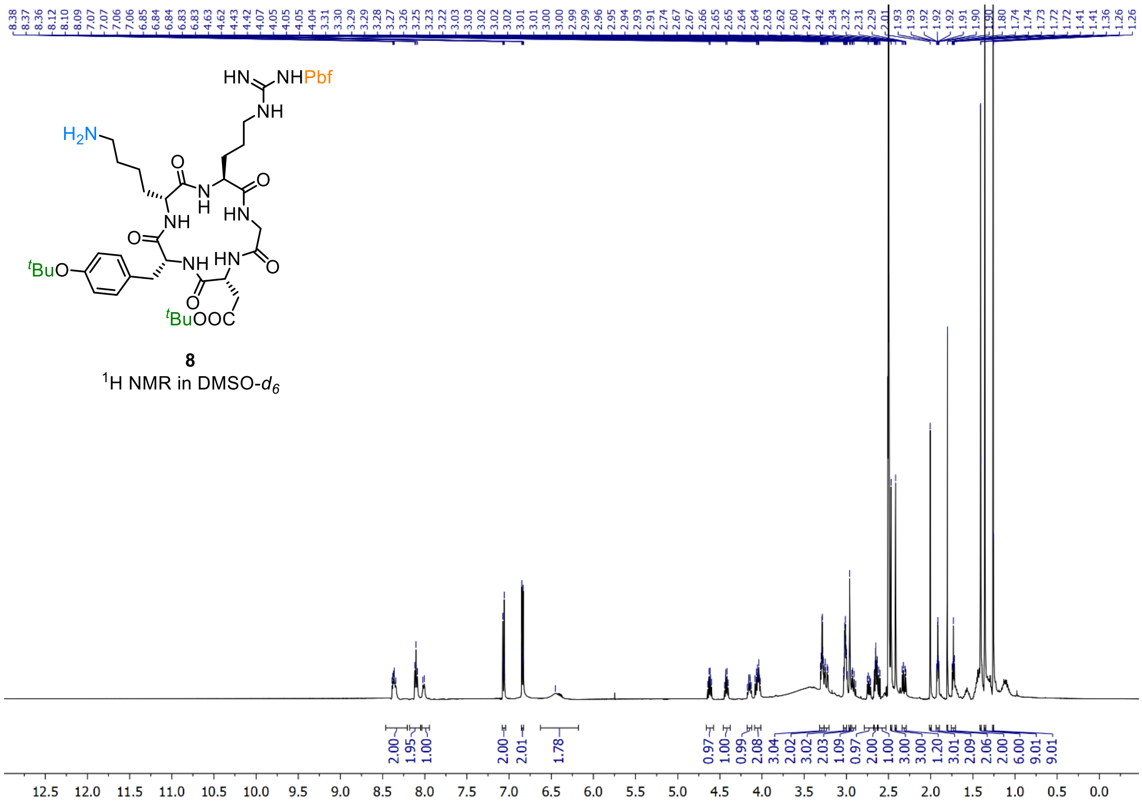

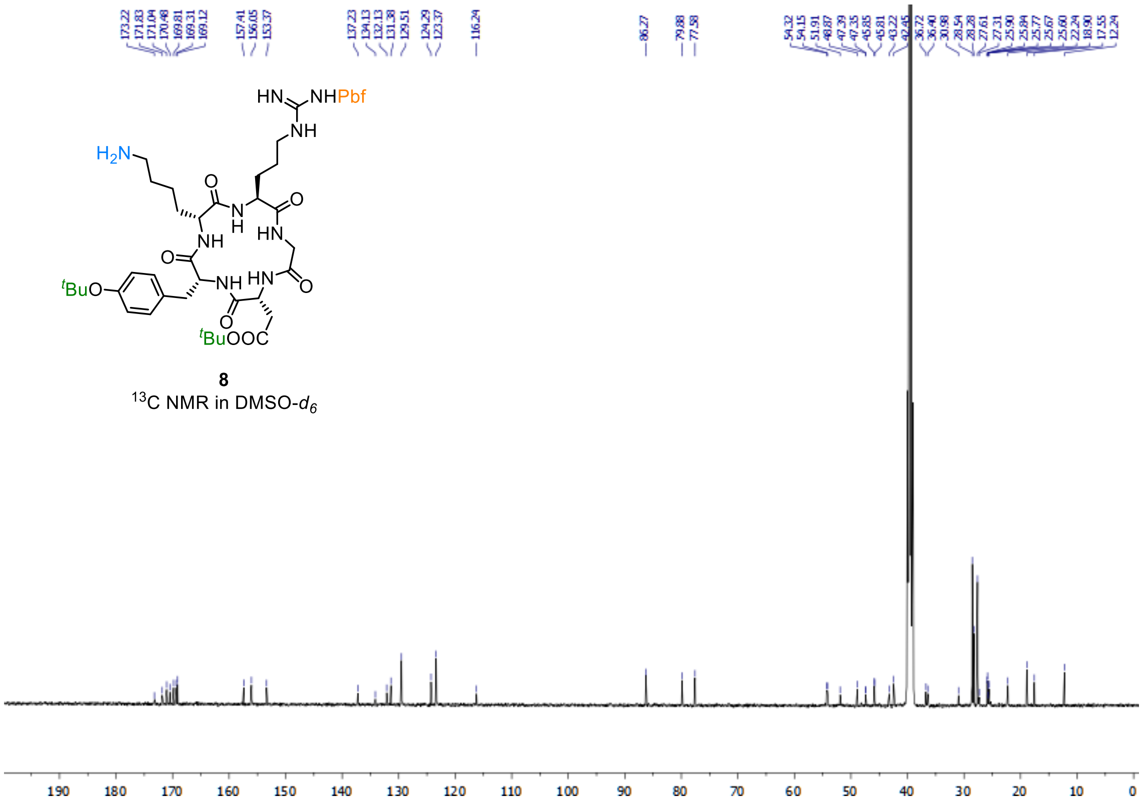

*Final cRGD-deferoxamine conjugate **9***

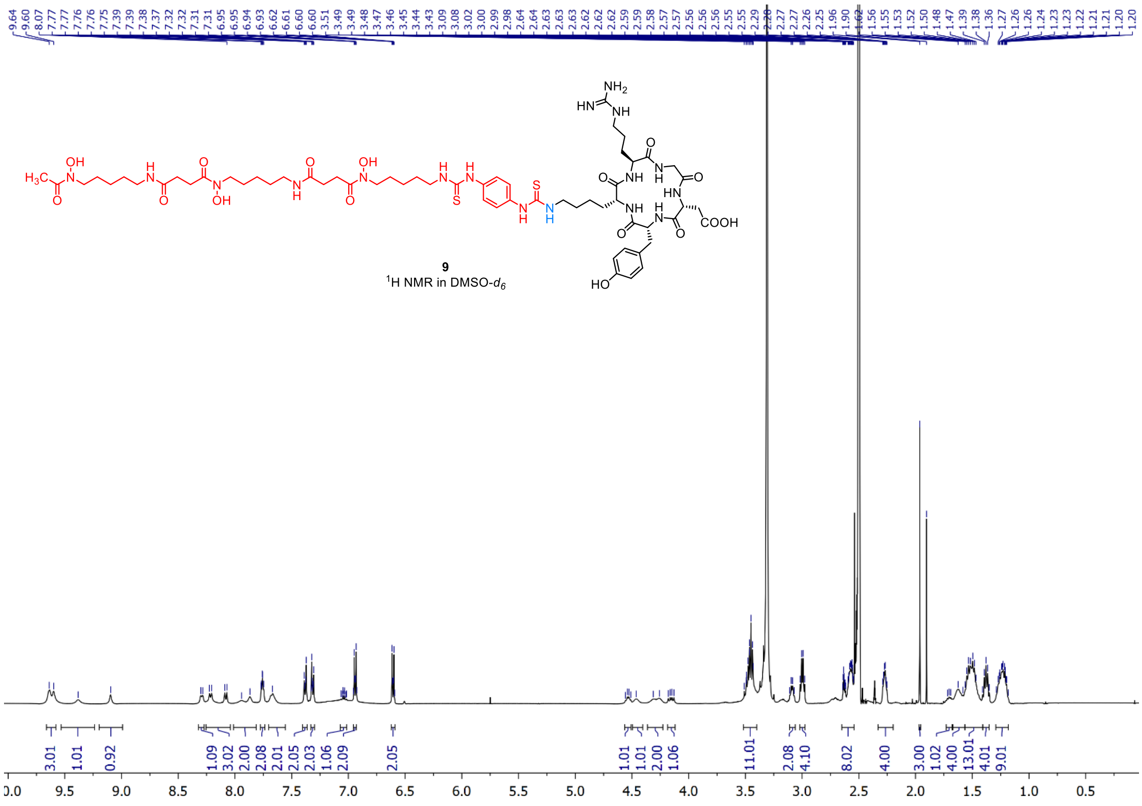

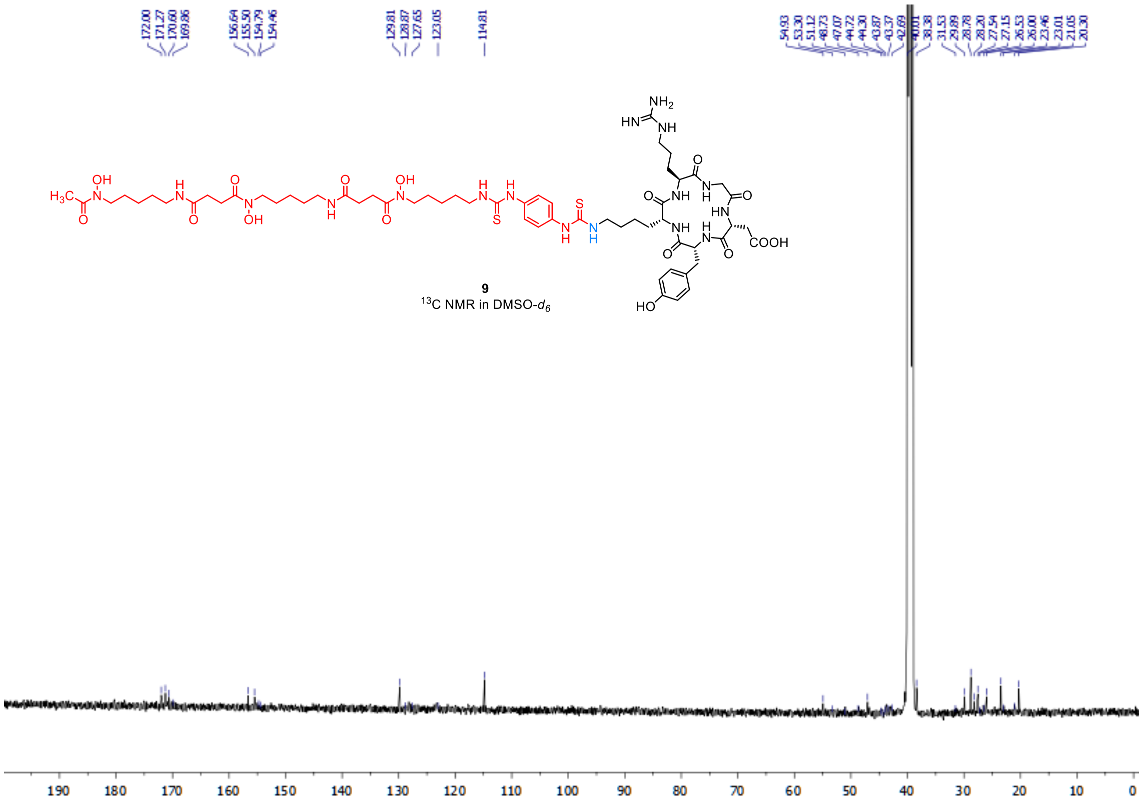

Supplement: Supplementary file 1 [file ijms-22-07391-s001.zip › ijms-1240751-supplementary.pdf]
